# A novel c.59 C > T variant of the *HSD17B10* gene as a possible cause of the neonatal form of HSD10 mitochondrial disease with hepatic dysfunction: a case report and review of the literature

**DOI:** 10.1186/s13023-024-03513-2

**Published:** 2025-03-07

**Authors:** Tao Jiang, Wenxian Ouyang, Haiyan Yang, Shuangjie Li

**Affiliations:** 1 Department of Hepatopathy Center, Hunan Children’s Hospital, Ziyuan Road 86th, Changsha, 410007 Hunan P. R. China; 2 Neurology Department, Hunan Children’s Hospital, Changsha, Hunan China

**Keywords:** HSD10 mitochondrial disease, *HSD17B10* gene, Variant, Cholestatic hepatitis, Metabolic disorder

## Abstract

**Background:**

Pathogenic *HSD17B10* gene variants cause HSD10 mitochondrial disease (HSD10 MD), which results in a wide spectrum of symptoms ranging from mild to severe. Typical symptoms include intellectual disability, choreoathetosis, cardiomyopathy, neurodegeneration, and abnormal behavior. This study investigated a novel c.59 C > T variant of the *HSD17B10* gene and the clinical phenotypic features of HSD10 MD (neonatal form) patients.

**Results:**

We describe a Chinese boy 2 months and 12 days old with intellectual disability, metabolic acidosis, hyperlactatemia, hypoglycemia, cholestatic hepatitis and myocardial enzyme levels, slightly elevated 2-methyl-3-hydroxybutyric acid (2M3HBA) levels and early death. Although full-length sequencing of the mitochondrial genome was normal, whole-exome sequencing of the proband and his parents revealed a novel de novo hemizygous variant, c.59 C > T (p.S20L), of the *HSD17B10* gene. Molecular dynamics simulation analysis and protein structural analysis suggested that the c.59 C > T (p.S20L) variant may disrupt the conformational stability of the protein. On the basis of the combined results of phenotypic analysis, molecular genetic analysis, protein structural analysis and molecular dynamics simulation analysis, this novel variant is currently considered a likely pathogenic variant. HSD10 MD (neonatal form) can lead to hepatic dysfunction.

**Conclusions:**

HSD10 MD (neonatal form) can lead to hepatic dysfunction. The de novo c.59 C > T *HSD17B10* variant suggested a neonatal form of the HSD10 mitochondrial disease phenotype in a patient 2 months and 12 days old, broadening the variant spectrum of *HSD17B10-*related disease.

## Introduction

HSD10 mitochondrial disease (OMIM 300438) was originally described as 2-methyl-3-hydroxybutyryl-l CoA dehydrogenase deficiency (MHBDD), a rare X-linked recessive genetic disorder. The multifunctional 17β-HSD10 protein is encoded by the *HSD17B10* gene. The protein is widely distributed in various tissues and organs, mainly the heart, liver and brain [1, 2]. Normal levels of 17β-HSD10 are essential for maintaining human health, especially normal cognitive function [3]. The 17β-HSD10 protein plays important roles in the metabolism of isoleucine and branched-chain fatty acids [3], neurosteroids, and sex steroid hormones [4].

As a component of the mitochondrial RNase P protein complex [5], 17β-HSD10 participates in mitochondrial tRNA processing and maturation and ultimately mitochondrial protein synthesis [6] and is essential for the structural and functional integrity of mitochondria [7]. Indeed, complete loss of 17β-HSD10 is incompatible with life. The clinical symptoms of patients with HSD10 MD are unrelated to the accumulation of toxic metabolites in the isoleucine pathway and the residual enzymatic activity of 17β-HSD10 [1]. The elevation of 2-methyl-3-hydroxybutyric acid (2M3HBA) and tiglylglycine (TG) in urine organic acid analysis is often helpful in the biochemical diagnosis of HSD10MD [6]. The pathology of HSD10 MD is thought to be caused by mitochondrial dysfunction [7–10]. Pathogenic variants of the *HSD17B10* gene cause HSD10 MD, whose clinical features are similar to those of severe mitochondrial diseases (progressive neurodegeneration, cardiomyopathy and metabolic disorders). Since the first report of HSD10 MD in 2000 [2], relatively few cases worldwide have been reported in the literature [6, 11–13].

Here, we report a novel variant of the *HSD17B10* gene (c.59 C > T) in a boy 2 months and 12 days old with a newly described aspect of the clinical phenotype of HSD10 MD, which manifested as mild developmental delay, metabolic disorders, hyperlactatemia, cholestatic hepatitis, elevated myocardial enzymes and 2M3HBA levels and early death. Through combining clinical analyses with molecular genetic analysis, protein structural analysis and molecular dynamics simulation analysis, we identified a novel c.59 C > T variant in HSD17B10 as the likely genetic cause of HSD10 MD in the present case. We found that the neonatal form of HSD10 disease can lead to hepatic dysfunction.

## Case report

The patient was a 72-day-old Chinese boy who was admitted to our hospital because of jaundice. He was small for gestational age as a newborn (birth weight 2 kg, G1P1, P3). Polypnea and jaundice were noted on the second day after birth. He was diagnosed with pneumonia, neonatal metabolic acidosis, hyperlactic acidemia and neonatal hyperbilirubinemia in the hospital. His parents were healthy. He presented with mild developmental delay, malnutrition (WT 3.6 kg, P3), yellow skin, yellow sclera, and hepatomegaly but normal myodynamia and muscular tension. Laboratory examination revealed cholestasis, elevated transaminase and myocardial enzymes, hypoglycemia, hyperlactinemia, metabolic acidosis and anemia (Table 1). His plasma amino acid analysis was normal (proline, 92.97 µmol/L [reference range, 72–293]; alanine, 326.49 µmol/L [reference range, 62.9–500]). Gas chromatography–mass spectrometry (GC–MS) of the urine sample revealed an increase in lactic acid (22.6; reference range, 0.0–13.0) and a slight increase in 2M3HBA (5.7; reference range, 0.0–4.0). The level of tiglylglycine was normal. Cardiac color Doppler ultrasound suggested a patent foramen ovale. Abdominal color Doppler ultrasound revealed a fine light spot in the liver and an enhanced echo. The patient was treated with reduced glutathione and ursodeoxycholic acid. The bilirubin level improved after treatment (Table 1). One month later (age 116 days), our patient had an upper respiratory infection and died while en route to the hospital.

**Table 1:** Changes and normal values of biochemical indicators in children

age	TBIL µmol/L	DBIL µmol/L	ALB g/L	GLO g/L	ALT IU/L	AST IU/L	γ-GGT IU/L	TBA µmol/L	LDH IU/L	CK U/L	CK-Mb U/L	LAC µmol/L	BS mmol/L
72 days	218.6	176.2	37.6	10.8	58.5	106.9	138.7	97.2	620	264	66.3	13.71	4.66
76 days	155.3	101.1	34.1	12.8	28.5	97	101	106.8	571	282.5	50.5	17.25	0.99
86 days	155.4	143.9	41.9	11.7	46.3	118.8	101.5	112.5	505	292	76.4	17.95	2.69
Normal range	3.4–17.0	0–6.0	35–55	20–35	0–40	0–40	0–50	0-9.67	0-450	38–174	0–24	0.5–2.44	3.9–6.1

TBIL = total bilirubin, DBIL = bilirubin direct, ALB = albumin, GLO = globulin, ALT = Alanine transaminase, AST = aspartate aminotransferase, γ-GGT = gamma-glutamyl transpeptidase, TBA = total bile acid, LDH = lactic dehydrogenase, CK = creatine kinase, CK-MB = Creatine kinase isoenzyme, LAC = lactic acid, BS = blood glucose

## Methods

### Patient and ethical considerations

The patient was managed at the Department of Hepatopathy Center, Hunan Children’s Hospital. Informed consent was obtained from the patient’s parents, and all clinical investigations were carried out in accordance with the Declaration of Helsinki [14]. This study was approved by the Medical Ethics Committee of Hunan Children’s Hospital (HCHLL-2022–120).

### Genetic analysis

A 2 mL sample of peripheral blood in EDTA anticoagulant tubes was extracted from the child and each of his parents, and whole-exome sequencing and a full-length PLUS test of the mitochondrial genome were performed. Genomic DNA was extracted from peripheral blood via a Solpure Blood DNA Kit (Magen) according to the manufacturer’s instructions. The genomic DNA was then fragmented with a Q800R sonicator (Qsonica) to generate 300–500 bp insert fragments. Paired-end libraries were prepared following the Illumina library preparation protocol. Custom-designed NimbleGen SeqCap probes (Roche NimbleGen, Madison, WI) were used for in-solution hybridization to enrich target sequences. The enriched DNA samples were indexed and sequenced on a NextSeq500 sequencer (Illumina, San Diego, California) with 100–150 cycles of single-end reads according to the manufacturer’s protocols. Primary data were generated in fastq format after image analysis, and base calling was conducted via the Illumina Pipeline. Sequence variants were annotated via population and literature databases, including the 1000 Genomes, dbSNP, GnomAD, ClinVar, HGMD and OMIM databases. The online software package was used to analyze the structure of the protein, predict the conserved domain and functional domain and perform multiple sequence alignment. Variant interpretation was performed according to the guidelines of the American College of Medical Genetics (ACMG) [15]. Full-length amplification and sequencing of the mitochondrial genome were performed according to our previous methods [16].

### Molecular dynamics simulation

The 3D structure of HSD17B10 was downloaded from the RCSB database (PDB ID: 1u7t). Chain B was used for visualization analysis and molecular dynamic simulation. The cofactor NAD + in the crystal structure was retained. The Mutagenesis Wizard in the PyMOL 1.7 package was used to construct the S20L mutant.

### Conservation analysis and protein schematic structures

Furthermore, we performed a conservation analysis of the four mutant amino acid sequences (mutation types in neonatal-type patients) by screening HSD17B10 orthologs against the NCBI HomoloGene database (https://www.ncbi.nlm.nih.gov/homologene/?term=) via the transcript NM_004493. The selected homologous amino acid sequences were downloaded and visualized with Ugeneui. Three-dimensional structural models of HSD17B10 were predicted with the AlphaFold tool (https://AlphaFold.ebi.ac.uk/). Protein structure images were generated via the PDB file and PyMOL. Hydrogen bonds in the proteins were identified via PyMOL to predict changes in mutant stability.

## Results

### Genetic analysis

**Fig. 1:**
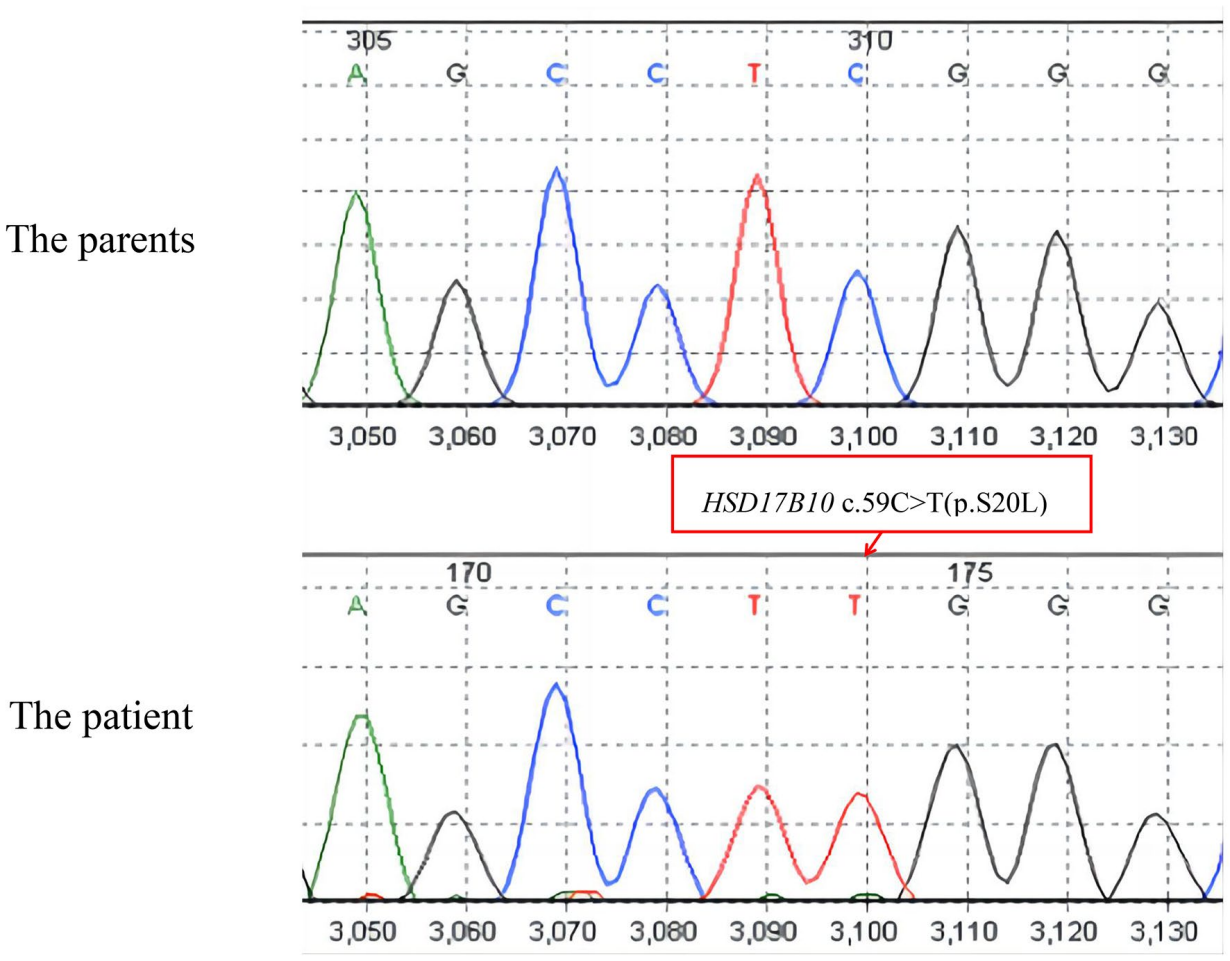
Results of whole-exome sequencing of the patient. The patient had a HSD17B10 c.59 C > T (p. S20L) mutation, which was de novo. His parents did not carry this mutation

A hemizygous variant of HSD17B10 was identified via whole-exome sequencing. The hemizygous mutation c.59 C > T (p.S20L) is a missense mutation. The C-to-T mutation at position 59 in the coding region of the gene results in a change in amino acid 20 from serine to leucine. The patient’s parents did not carry this mutation (Fig. 1). This variant has not been recorded in the HGMD, PubMed, ClinVar or other databases and has not been reported in related clinical cases. The mutation was determined to be de novo, with a low frequency (less than 0.001) in our reference population gene database. The region where the variation occurs is an important part of the protein, and the amino acid sequence is highly conserved in different species. Computer-aided analysis predicted that this variant is likely to affect protein structure/function. In summary, on the basis of the clinical manifestations of the patient and family analysis, the mutation was classified as “class 2—possibly pathogenic” according to the ACMG standards(PM). Full-length sequencing of the mitochondrial genome of peripheral blood revealed normal results.

### Molecular dynamics simulation

**Fig. 2:**
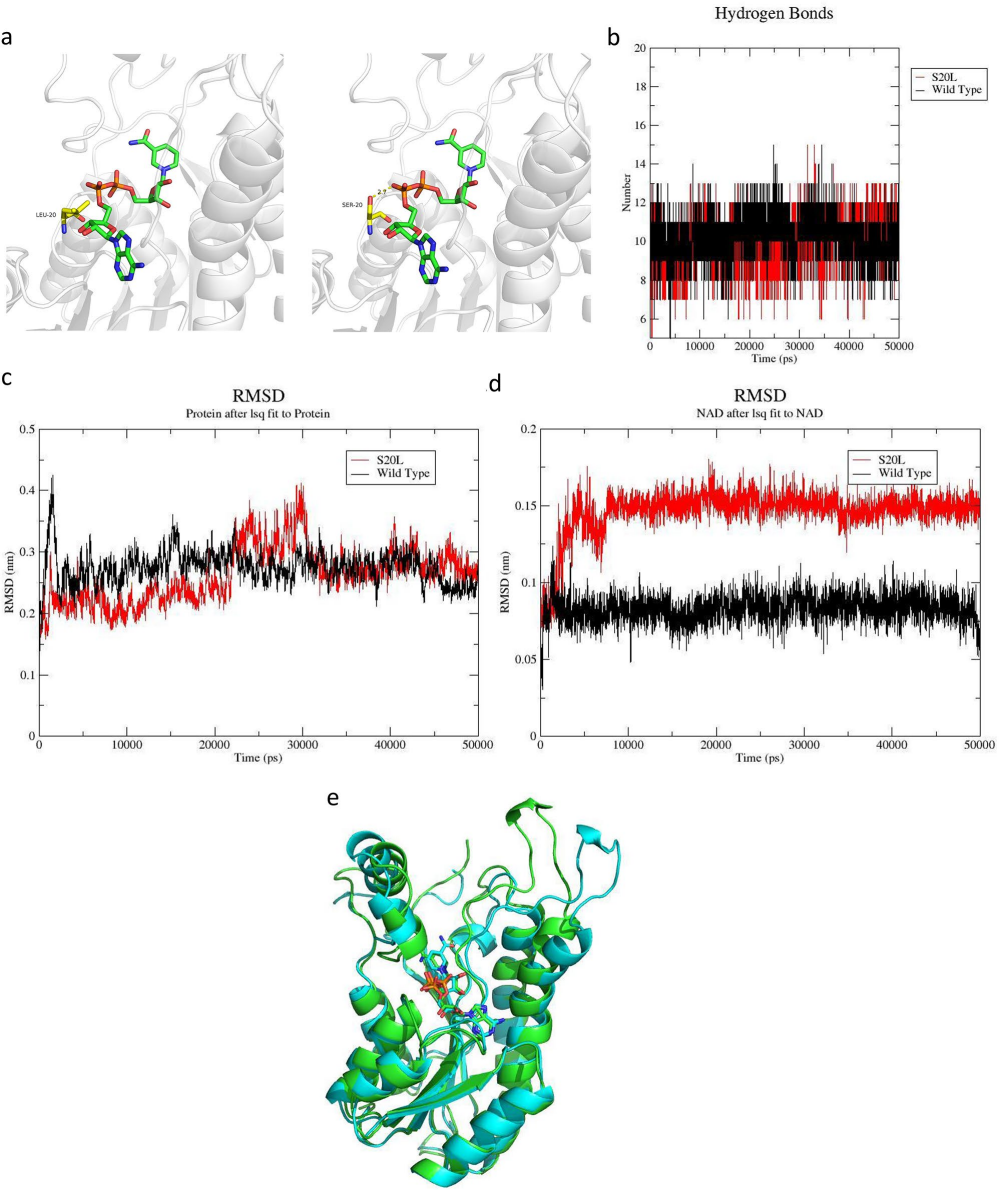
Molecular Dynamics Simulation analysis of the c.59 C > T (p.S20L) variant. NAD + showed a stronger binding affinity for the wild-type protein than for the mutant protein

In the wild-type protein, Ser20 forms a strong hydrogen bond with the phosphate group of NAD+, but in the S20L mutant, this H-bond cannot be formed by the side chain of Leu (Fig. 2a). We speculated that this H-bond is highly important for the stable binding of NAD+. Without this H-bond, NAD + may not bind very well, thus influencing the catalytic activity of the enzyme. The number of hydrogen bonds between NAD + and the wild-type enzyme was clearly greater than that between NAD + and the mutant enzyme during the 50 ns molecular dynamic simulation (Fig. 2b). These findings indicated that NAD + could bind more stably to the wild-type enzyme than to the S20L mutant enzyme. We used the RMSD to determine the conformational fluctuations of the protein (or ligand) and NAD+. Compared with the wild-type enzyme, the S20L mutant had a much greater RMSD (Fig. 2c). NAD + bound to the mutant protein had a much greater RMSD value than that bound to the wild-type protein (Fig. 2d). This result indicated that NAD + bound strongly to the WT receptor protein. Next, we used the MMGBSA method to calculate the binding free energy between NAD + and the wild-type receptor protein (enzyme) or the mutant protein on the basis of the molecular dynamics trajectory. NAD + showed a stronger binding affinity for the wild-type protein than for the mutant protein (binding free energy, -83 vs. -62 kcal/mol). Figure 2e shows the structural alignment of the wild-type and mutant proteins after the 50 ns molecular dynamic run. The mutant protein has a more expanded conformation than the wild-type protein does, which is consistent with its high RMSD.

### Conservation analysis and protein schematic structures

**Fig. 3:**
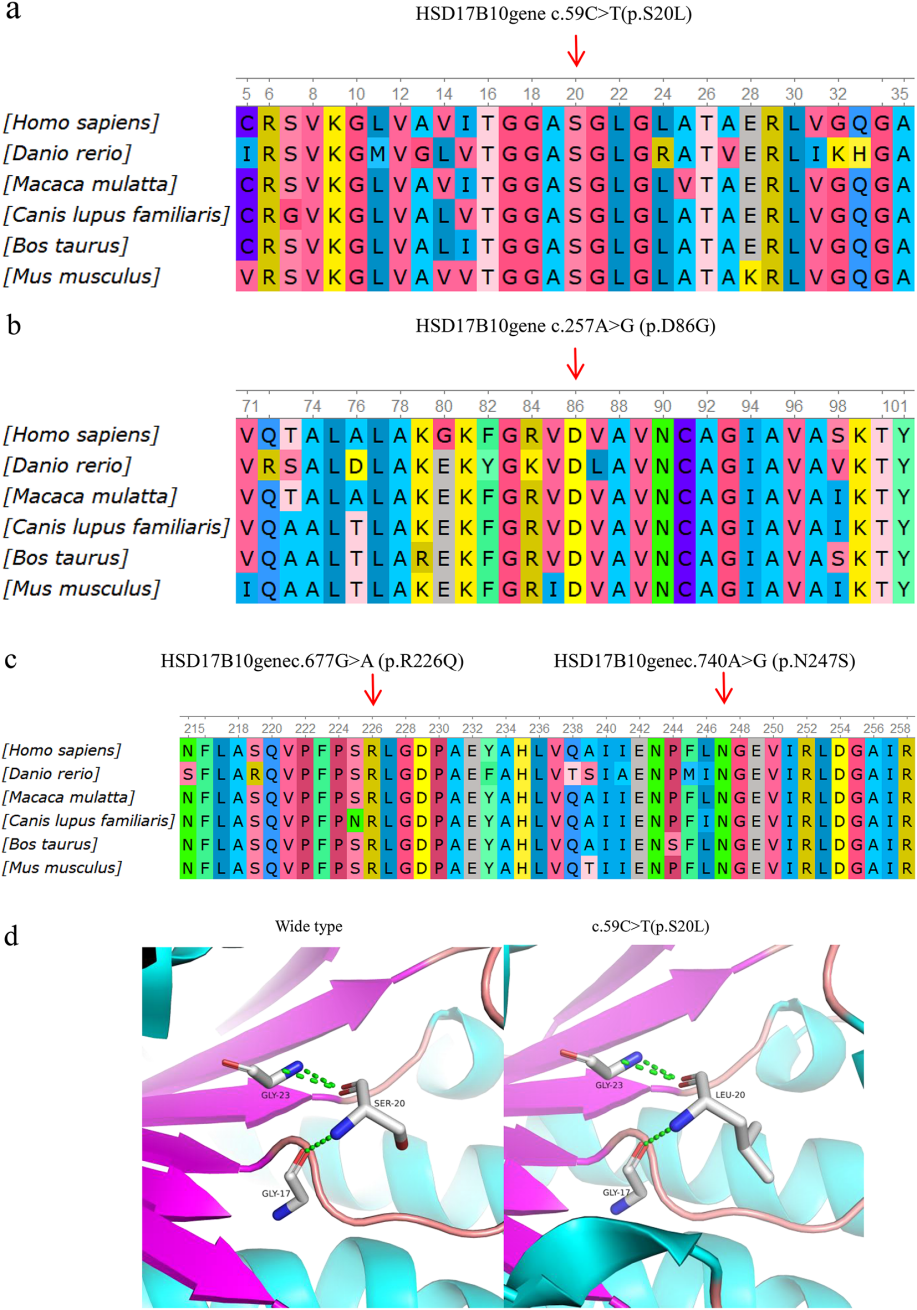

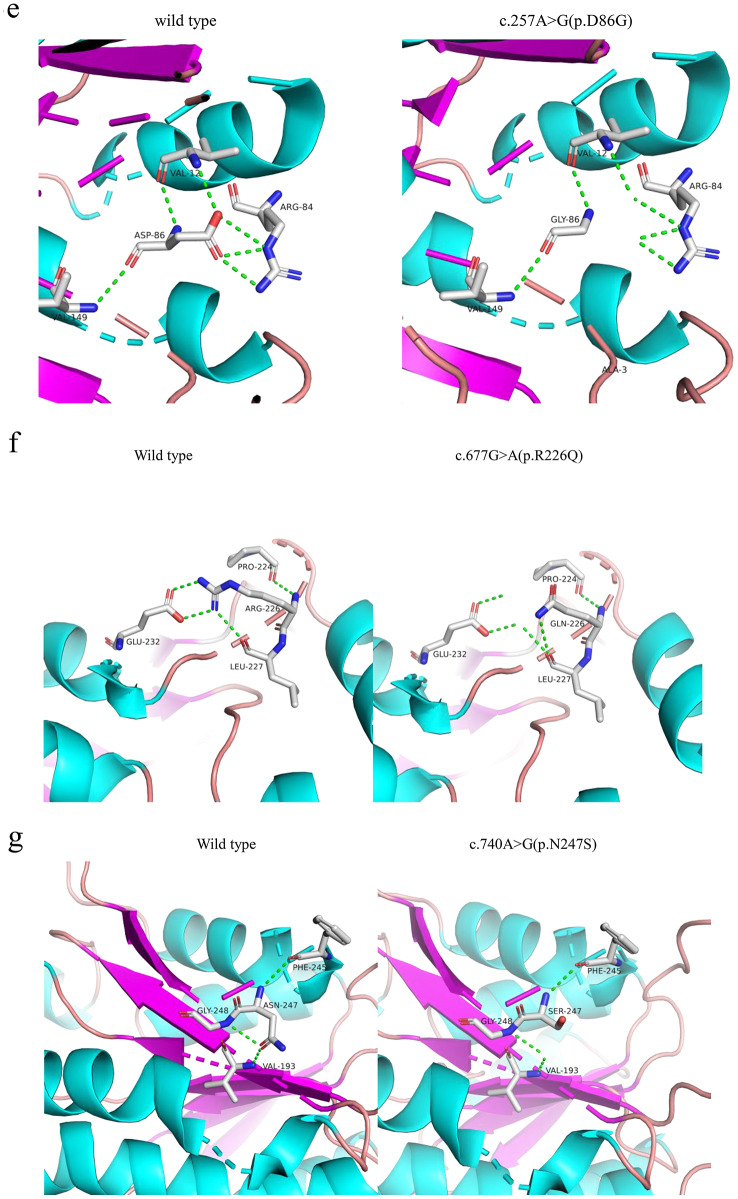
Conservation analysis and protein schematic structures of four mutant amino acid. Four mutations affect the structure of the HSD17B10 protein

The mutation types in neonatal-type patients were c.59 C > T (p.S20L), c.740 A > G (p.N247S), c.677G > A (p.R226Q) and c.257 A > G (p.D86G). A conservation analysis of the four mutant amino acid sequences is shown in Fig. 3a-Fig. 3c. The amino acids N247, S20, and R226 are close to the amino acid D86, which was mutated in a clinically severely affected patient with preserved MHBD enzymatic function [7]. Structural analysis of the Mut-HSD17B10 protein revealed that the identified mutation (p.S20L) changes the hydrophilic amino acid threonine to the hydrophobic amino acid leucine (Fig. 3d). The mutation p.D86G disrupts the intermolecular hydrogen bond between amino acid 86 and amino acid 84 (Fig. 3e). The p.R226Q mutation breaks the intermolecular hydrogen bond between amino acids 226 and 232 and results in the formation of a new hydrogen bond with amino acid 227 (Fig. 3f). The mutation p.N247S disrupts the intermolecular hydrogen bond between amino acid 247 and amino acid 193 (Fig. 3g).

## Discussion

The age of onset of HSD10 disease can range from the newborn period to early childhood. The most severely affected patients are males (hemizygous), whereas heterozygous females are generally asymptomatic or have only mild symptoms [1]. HSD10 disease is characterized by progressive neurodegeneration and includes intellectual disability, language defects, bradykinesia, ataxia, epilepsy, visual and auditory disorders, hypotonia, cardiomyopathy and metabolic disorders. There are four clinical types of HSD10 disease: neonatal, infantile, juvenile and atypical/asymptomatic [1]. The most common type is the infantile form. The clinical features of our patient included mild developmental delay, metabolic acidosis, hyperlactatemia, hypoglycemia, cholestatic hepatitis and higher than normal myocardial enzyme levels; slightly elevated 2M3HBA levels; and early death. Whole-exome sequencing of the proband and his parents revealed a novel de novo heterozygous variant, c.59 C > T (p.S20L), of the *HSD17B10* gene. Combined with the highly characteristic clinical phenotype and molecular genetic analyses, HSD10 MD (neonatal form) was a likely diagnosis.

Hepatic involvement is a common feature of early-onset mitochondrial disease [17]. Genetically confirmed mitochondrial disease was observed in 17% of children who presented with acute liver failure before the age of 2 years [18, 19]. HSD10 disease manifests mainly in the central nervous system. The liver and kidneys are not usually affected by the infantile form [1]. Few patients with the neonatal form of HSD10 MD have been reported to date (Table 2). Two patients with the neonatal form had hepatic dysfunction [8, 10]. Chatfield et al. [8] reported infants with hepatomegaly. The histological features of the patients included micro- and macrovesicular steatohepatitis and disrupted mitochondrial architecture with a strongly increased number of mitochondria and abnormal cristae structure. Mitochondrial respiration and assembly are disrupted in HSD10 disease. Our patient also had hepatic dysfunction. Genetic tests did not reveal any other variants with the potential to cause mitochondrial disease or liver disease. Thus, the incidence of hepatic dysfunction in neonates with HSD10 MD is relatively high (50%). If patients have hepatic dysfunction and metabolic derangement during the neonatal period, we should consider that they may have HSD10 disease.

**Table 2:** Genotypes and phenotypes summary in neonatal form of HSD10 MD

	Mutation	Sex	Onset	Clinical feature	Brain MRI	Family history	Present status (age)
Patient 1 (this report)	c.59C > T(p.S20L)	M	2d	development retardation, metabolic acidosis, hyperlactatemia, hypoglycemia, Cholestatic hepatitis, elevated myocardial enzyme.	NA	Normal parents	Dead (3m)
Patient 2^[17]^	c.740A > G (p.N247S)	M	1d	Metabolic acidosis, hypoglycemia, hypotonia, cyanosis, cardiomegalia, hyperlactatemia and hyperlactaturia	NA	sister is patient, Normal mother	Dead (2 m)
Patient 3^[10]^	c.677G > A **(p.R226Q)**	M	1 d	Developmental regression, metabolic acidosis, hypoglycemia, anemia, hyperamoniemia, thrombopenia, cuagulopathy, hepatic dysfunction, myoclonus, seizures, hypertrophic miocardiopathy	Isquemic lesions in nucleus	Normal mother	Dead (7 m)
Patient 4^[7]^	c.257A > G(p.D86G)	M	NA	Neurological development, progressive hypertrophic cardiomyopathy	NA	NA	Dead (7 m)
Patient 5^[8]^	c.740A > G(p.N247S)	M	1d	mildly encephalopathic, hyperlactatemia, hyperlactaturia, hyperammonemia, feeding difficulties, PDA(patent ductus arteriosus), anemia, thrombocytopenia,, elevated transaminases, cuagulopathy	restricted diffusion in the perirolandic white matter	NA	Dead (6 m)
Patient 6^[18]^	c.677G > A(p.Arg226Gln)	M	1d	Polypnea, moan, hypoglycemia, hyperlactatemia, psychomotor retardation	a slightly deeper sulci in the cerebral hemisphere	NA	abandoned the treatment

NA: not available

The 17β-HSD10 protein is encoded by the *HSD17B10* gene, which maps to chromosome Xp11.2 and consists of 6 exons. In total, 16 different missense variants and a splicing mutation are reported to cause HSD10 MD [4, 11, 20–24]. The mutation c.388 C > T (p.R130C) is the most frequent variant [12]. At present, the reported mutation types in neonatal patients include c.740 A > G (p.N247S), c.677G > A (p.R226Q) and c.257 A > G (p.D86G). This study also revealed a new mutation that causes neonatal-type HSD10 disease. Protein structural analysis revealed that may impact the local secondary structure and molecular function of the protein. The mutated amino acid D86 was found in a clinically severely affected child and caused severe disruption of mitochondrial morphology. The amino acid Q165 was observed in a child who was clinically mildly affected but completely deficient in MHBD enzyme activity [7]. The amino acids N247 [8], S20, and R226 are close to the amino acid D86 and away from the amino acid Q165. These mutations may increase the likelihood of mitochondrial dysfunction, which can lead to disease. Furthermore, we classified the missense variant c.59 C > T as a likely pathogenic candidate for causing this proband’s clinical manifestations for the following reasons. First, the clinical features of the patient were consistent with those of patients with the neonatal form of HSD10 MD. Second, this variant is not listed in the HGMD, PubMed, ClinVar or other databases or the related literature. The ACMG variant classification guidelines classify patients as “class 2—possibly pathogenic”. Third, molecular dynamics simulation and protein structural analysis revealed that the mutation may disrupt the conformational stability of the protein. The apparent impact of this variant on the conformational stability of the protein is relevant to the occurrence of clinical disease.

This study has several limitations. Brain MRI was not performed. No studies to determine the effect of the mutation on protein expression were performed. Owing to the early death of our patient, functional verification was not carried out. The same mutation can lead to different clinical manifestations. Owing to the small number of cases reported thus far, additional evidence is needed for a clear genotype–phenotype correlation.

There is no effective therapy for HSD10 MD. Cardiac failure, Kussmaul breathing, and multiple-organ dysfunction may be induced by mild infection [8]. Our patient had an upper respiratory tract infection before death. Therefore, rapid intervention in patients with acute infection is highly important.

In conclusion, HSD10 MD (neonatal form) can lead to hepatic dysfunction. Our results add evidence that the de novo variant c.59 C > T (p.S20L) may have caused HSD10 MD (neonatal form) in this patient seen at 2 months and 12 days old, broadening the spectrum of *HSD17B10*-related disease.

## Data Availability

PRO-Seq data were deposited into ClinVar under accession number SUB14700544 and are available at the following URL: https://www.ncbi.nlm.nih.gov/clinvar/variation/3339520/?oq=SUB14700544&m=NM_004493.3(HSD17B10):c.59 C%3ET%20(p.Ser20Leu).

## Abbreviations

2M3HBA 2: Methyl-3-hydroxybutyric acid
17ß-HSD10: 17ß-hydroxysteroid dehydrogenase type 10
HSD10MD: HSD10 mitochondrial disease
MD: Mitochondrial disease
RMSD: Root-mean-square deviation
MMGBSA: Molecular mechanics-generalized born surface area
TG: Tiglylglycine
